# Entropy and Contrast Enhancement of Infrared Thermal Images Using the Multiscale Top-Hat Transform

**DOI:** 10.3390/e21030244

**Published:** 2019-03-04

**Authors:** Julio César Mello Román, José Luis Vázquez Noguera, Horacio Legal-Ayala, Diego P. Pinto-Roa, Santiago Gomez-Guerrero, Miguel García Torres

**Affiliations:** 1 Facultad Politécnica, Universidad Nacional de Asunción, San Lorenzo 2160, Paraguay; 2 Division of Computer Science, Universidad Pablo de Olavide, ES-41013 Seville, Spain

**Keywords:** discrete entropy, infrared images, low contrast, multiscale top-hat transform

## Abstract

Discrete entropy is used to measure the content of an image, where a higher value indicates an image with richer details. Infrared images are capable of revealing important hidden targets. The disadvantage of this type of image is that their low contrast and level of detail are not consistent with human visual perception. These problems can be caused by variations of the environment or by limitations of the cameras that capture the images. In this work we propose a method that improves the details of infrared images, increasing their entropy, preserving their natural appearance, and enhancing contrast. The proposed method extracts multiple features of brightness and darkness from the infrared image. This is done by means of the multiscale top-hat transform. To improve the infrared image, multiple scales are added to the bright areas and multiple areas of darkness are subtracted. The method was tested with 450 infrared thermal images from a public database. Evaluation of the experimental results shows that the proposed method improves the details of the image by increasing entropy, also preserving natural appearance and enhancing the contrast of infrared thermal images.

## 1. Introduction

Thermal infrared imaging (TII) is emerging as a powerful and non-invasive tool to accurately evaluate the thermal distribution of a body. TII is based on the physical phenomenon that all bodies above absolute zero emit thermal radiation. The intensity and spectral distribution of emitted radiation depend on the temperature, and its detection allows the creation of a thermal map of temperature distribution. TII uses the thermal radiation to create an image similar to visible light imaging. However, the use of this thermal radiation presents advantages over visible light in extreme situations since it can provide valuable information from an environment independent of the quality of the environmental light source, as is the case in foggy conditions or darkness, where TII can detect the presence of individuals, objects, or animals [1,2]. This feature makes the utilization TII very competitive to traditional methods in different fields as security, engineering, ecology, etc. [1,2,3].

Despite the advantages of TII, in some scenarios images may present low contrast, as well as low-level and blur details. These issues are due to facts such as limitations of the cameras with which the images are captured, conditions in the environment, etc. Therefore, contrast enhancement techniques may yield higher image details [4,5].

Many algorithms currently exist that enhance the contrast of infrared images. Histogram-based algorithms are widely used to enhance the brightness areas of an infrared image [6,7,8,9]. One of the most popular methods is the Histogram Equalization (HE). However, in the process of enhancing an image, HE drastically changes the average brightness of the image, resulting in loss of information and visually deteriorated images [10]. The HE variants cause the same problems, but to a lesser extent. Hence, global histogram-based algorithms cannot improve image entropy [11,12,13,14,15].

Other strategies for improving thermal infrared image are based on mathematical morphology. These are widely used to enhance contrast, improve details and edges, suppress noise, and enhance small targets [4,5,16,17,18,19,20,21,22]. However, the technique has some problems associated with the shape and size of the structuring element. In order to solve this problem, proposals have been presented where, in the basic operations of mathematical morphology, two structuring elements of equal sizes and different shapes are used [5,21,23]. Strategies have also been used within multiscale schemes, such as sequential toggle operators, to achieve improvements in infrared images [4,22,24,25].

The top-hat transform is one of the most used operations of mathematical morphology. Image enhancement by top-hat transform consists of adding bright areas and subtracting dark areas from the original image [26,27,28]. To improve the performance of top-hat transform, it is normally used in a multiscale scheme [29]. The multiscale top-hat transform can extract multiple useful features from the image, which are then used to enhance the infrared image. The multiscale top-hat transform scheme is widely used to make improvements in different types of grayscale images [26,30]. For example, it has been used to enhance retinal images [31], ultrasound images [32], and infrared images [16,22,33]. It has also been used in applications such as visible and infrared image fusion [34,35,36], image segmentation [37], and detection of small objects [21,22,38].

In the literature, the results obtained by infrared image enhancement algorithms based on multiscale mathematical morphology are generally evaluated using the following metrics: Peak Signal-to-Noise Ratio (PSNR) [21,30], which measures distortion in the improved images; and linear index of fuzziness (γ) measure [4,5,20,33], which quantifies the improvement in blurriness of infrared images. For the results of this work it is also of utmost importance to quantify the richness of the details of the infrared image by means of its entropy [39,40], contrast enhancement [16] to differentiate the objects from their background, and the mean brightness [11], which will tell us if the resulting image maintains its naturalness after the process of enhancement.

In this article we propose a new method based on the multiscale top-hat transform. Two geometrically proportional and flat structuring elements are used in top-hat operations [16]. The method improves the details of infrared images by increasing their global entropy. It also introduces less distortion, preserves natural brightness, and enhances contrast in the resulting thermal infrared images. In the proposed method, first the two structuring elements are selected to improve the performance of the multiscale scheme. It then extracts the light and dark areas of the image on multiple scales, and after that it sums and weighs the light and dark areas obtained. Finally, the infrared thermal image is enhanced by adding the bright regions and subtracting the dark regions.

The contributions of this work are: (1) proposing the top-hat transform by using two structuring elements of different sizes; (2) a new algorithm for improving entropy and contrast in TTI based on the multiscale top-hat transform.

The article is structured as follows: Section 2 presents the preliminary concepts of entropy and contrast, Section 3 presents the proposed method to improve the TII based on the multiscale top-hat transform, Section 4 shows the experimental results, and Section 5 concludes with the main contributions of the work.

## 2. Entropy and Contrast in Digital Images

TII often presents problems at the time of capture, such as poor details and low contrast. When you want to solve the above problems by means of strategies to improve the image, other types of inconvenience usually appear; for instance, loss of detail and naturalness in the image.

Entropy [39,40,41,42,43] quantifies the information content of the image. It describes how much uncertainty or randomness there is in an image. The more information the image contains, the better its quality. In [44], Wang et al. propose a method based on fractional Fourier entropy map, multilayer perceptron, and Jaya algorithm in multiple sclerosis identification. In [45], Zhang et al. propose a smart detection method for abnormal breasts in digital mammography. In this case, fractional Fourier entropy was employed to extract global features. In [46], Lee et al. investigate a framework for expressing visual information in bits termed visual entropy, based on information theory.

The entropy (E) referred to here is Shannon’s entropy. In the field of information theory, entropy, also called entropy of information and Shannon’s entropy, measures the uncertainty of a source of information [47]. Shannon’s entropy is defined as:

(1) $$E\left(I\right)=-\sum_{k=0}^{L-1}p\left(k\right)log_{2}\left(p\left(k\right)\right),$$

where *I* is the original image, p(k) is the probability of occurrence of the value *k* in the image *I*, and $L=2^{q}$ indicates the number of different gray levels. E(I) is a convenient notation for the entropy of an image, and should not be interpreted here as a mathematical expectation since *I* is not a random variable. It is not difficult to prove that if *q* is the number of bits representing each pixel in the image, then E(I)∈[0,q]; for this work q=8 for infrared thermal images in gray scale.

**Figure 1:**
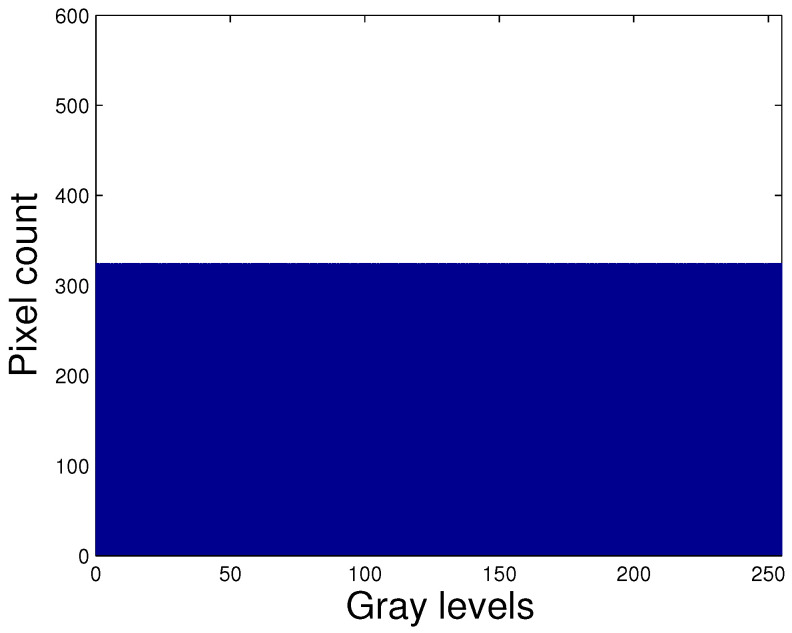
Histogram with uniform distribution.

In Figure 1 we can observe the histogram of an 8-bit image (histogram with uniform distribution). In this case the entropy has maximum value, i.e., the entropy has a value equal to 8. This happens when the probabilities of all possible results are equal. Also, it can be seen that the histogram uses all the available dynamic range, that is to say in the histogram we visualize all the values of intensity in the range [0,255]. Minimal entropy happens when the result is a certainty and its value is zero. In image processing, discrete entropy is a measure of the number of bits required to encode image data [41]. The higher the value of the entropy, the more detailed the image will be.

Contrast is defined as the difference between the light and dark areas of the image. The higher the variance of gray intensities, the higher the contrast. When the difference between the maximum and minimum intensities of an image is very small, the image has low entropy and poor contrast. Niu et al. [48] introduce a contrast enhancement algorithm of tone-preserving entropy maximization. Yoo et al. [10] propose an image enhancement method called MEDHS (Maximum Entropy Distribution based Histogram Specification), which uses the Gaussian distribution to maximize the entropy and preserve the mean brightness.

Unlike the methods mentioned above, in this work we propose a new method based on mathematical morphology. This method increases the global entropy and contrast, improving the details of the TII.

**Figure 2:**
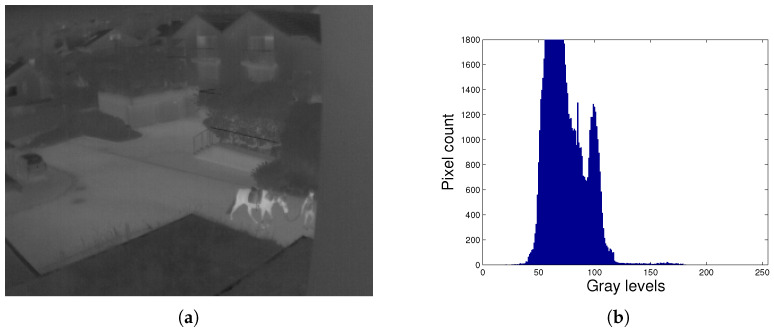
Thermal infrared image. (**a**) Original TII; (**b**) Histogram of TII.

In Figure 2 we can see the infrared thermal image with its associated histogram. Observing the histogram of the image, we can see that it does not effectively use the whole range of available intensity values. This indicates that the image has poor entropy and low contrast. When calculating Shannon’s entropy (Equation (1)) we can see that it has a value of E=6.008.

**Figure 3:**
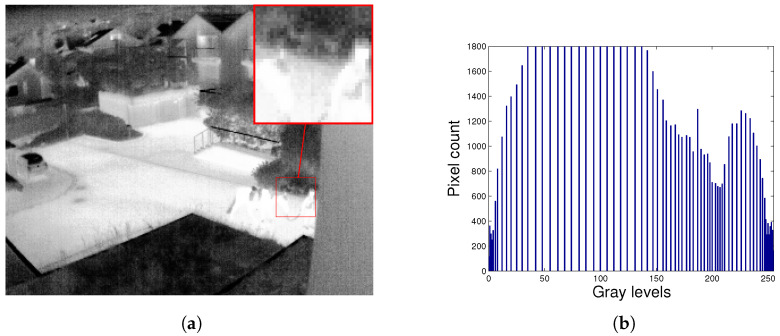
Loss of information with enhanced contrast. (**a**) TII enhanced with HE; (**b**) Histogram of the TII enhanced with HE.

As an example, Figure 3 shows the thermal infrared image (TII) obtained with the HE algorithm and its histogram. The HE method enhanced the contrast of the TII by making it brighter. In the histogram of the improved image we can visualize that the intensities are redistributed towards the available extreme values, leaving many holes. However, the method did not improve Shannon’s entropy, obtaining a value of E=5.933, which is less than the entropy of the unprocessed Figure 2a. Visually it is observed in Figure 3a that there is a loss in details, for example it is not possible to differentiate well the horse from the person.

To solve the problem of improving the image without incurring in a loss of the details and the mean brightness of the image, we will make a detailed description of the proposed method based on multiscale mathematical morphology in the following section.

## 3. Enhancement of Thermal Infrared Images

The top-hat transform is one of the most used operations of mathematical morphology to obtain improvements in the TII [4,5,16,20,21,22]. Two structuring elements of proportional sizes, equal shapes and planes, will be used to improve the performance of the top-hat transform [16].

### 3.1. Classic Top-Hat Transform

The top-hat transform is a composite operation of mathematical morphology; it is defined from other morphological operations, namely erosion, dilation, opening, and closing.

The morphological operations of dilation and erosion of I(u,v) for B(s,t), denoted by (I⊕B) and (I⊖B), are defined as follows [27,49]:

(2) $$\begin{array}{c} \left(I⊕B\right)\left(u,v\right)=\max_{(s,t)\in I}\left\{I\left(u+s,v+t\right)+B\left(s,t\right)\right\}, \end{array}$$

(3) $$\begin{array}{c} \left(I⊖B\right)\left(u,v\right)=\min_{(s,t)\in B}\left\{I\left(u+s,v+t\right)-B\left(s,t\right)\right\}. \end{array}$$

where *I* is the original infrared thermal image whose pixels are represented for all (u,v) spatial coordinates and *B* is the structuring element whose spatial coordinates are represented by (s,t).

The opening (I∘B) and closing (I•B) morphological operations of I(u,v) for B(s,t) are defined from the dilation and erosion operations as follows [27,49]:

(4) I∘B=(I⊖B)⊕B,

(5) I•B=(I⊕B)⊖B.

The top-hat transform morphological operation [27] is defined from the morphological opening and closing. White Top-Hat (WTH) is the top-hat transform through opening, Black Top-Hat (BTH) is the top-hat transform through closing. WTH gets the bright areas and BTH gets the dark areas lost in the opening and closing operations. Both transforms are defined as follows:

(6) WTH=I−(I∘B)=I−((I⊖B)⊕B),

(7) BTH=(I•B)−I=((I⊕B)⊖B)−I.

### 3.2. Modified Top-Hat Transform

The classical top-hat transform is characterised by the use of a single structuring element. This makes its image processing performance inefficient [29]. To improve the performance of the top-hat transform it is proposed to use two structuring elements, whose characteristics will be proportional geometry and flat [16]. The Modified White Top-Hat (MWTH) and the Modified Black Top-Hat (MBTH) transforms will be used for image improvement within the scheme of multiscale top-hat transform.

Let the structuring elements be *G* and $G^{\prime }$ geometrically proportional and flat. Then, the top-hat transform that we will use in the multiscale scheme is defined as follows:

(8) $$MWTH=I-(\left(I⊖G\right)⊕G^{\prime }),$$

(9) $$MBTH=(\left(I⊕G\right)⊖G^{\prime })-I.$$

Note that if $G=G^{\prime }$, then Equations (8) and (9) are equal to Equations (6) and (7). Therefore, the classical top-hat transform is a particular case of the modified top-hat transform. In [16], Román et al. show that the modified top-hat transform improves thermal images, enhancing the contrast, preserving the details and introducing less distortion.

### 3.3. How Entropy is Changed by Top-Hat Transform

The Shannon entropy depends on both (a) the number of distinct values exhibiting a positive frequency, and (b) how uneven the density function is, compared with a discrete uniform distribution.

The top-hat transform, working within a local region of the image, often generates new values of grey, thereby causing a small to moderate increase in the entropy value of the region. This occurs because Equations (8) and (9) can induce one or more new levels of grey when the logic is executed.

When one new level of grey *h* is added by the algorithm to a region being worked, it replaces another value *g* at certain spatial position. There are two possibilities: either

- The old value *g* was unique in the region, with a count of 1, hence it disappears from the region and is replaced by value *h*. No change in entropy occurs because in the old *g* bin of the histogram the count of 1 becomes 0, and in the new *h* bin the count of 0 becomes 1; or
- The old value *g* existed in *k* > 1 pixels in the region. In this case the count in the *g* bin decreases to k−1, and the count in the *h* bin increases to 1. The following Lemma shows that this change in the histogram increases the region’s entropy.

**Lemma** **1.** *Consider a rectangular region of m pixels in an image. Let* H(X) *be the original entropy of the grey scale X in use. Suppose that grey level g appears in k pixels of the original image and grey level h does not appear. Further, suppose that an image transformation replaces grey level g with grey level h at certain pixel of the rectangular region. Then the entropy of the transformed region increases to*

(10) $$H^{\prime }\left(X\right)=H\left(X\right)-p_{g}\;\left[\left(1-\varepsilon \right)\;\log\left(1-\varepsilon \right)+\varepsilon \;\log\left(\varepsilon \right)\right]$$

*where the value of ε is 1/m, the inverse of the number of pixels in the region.*

**Proof.**  Without loss of generality assume 255 levels of grey; thus both *g* and *h* are integers in {1,…,255}. As the sum of probabilities before and after the transformation equals 1, the increase in $p_{h}$ occurs at the expense of a decrease in $p_{g}$; that is, $p_{h}$ increases from 0 to $\varepsilon p_{g}$ and $p_{g}$ decreases to $\left(1-\varepsilon \right)p_{g}$.

For the region under consideration, Equation (1) can be written as

$$H\left(X\right)=-\sum_{i=0}^{255}p_{i}\log\left(p_{i}\right)=-\left[p_{0}\log\left(p_{0}\right)+\cdots +p_{255}\log\left(p_{255}\right)\right].$$

After transformation, the probability corresponding to level *g* is broken down in two: a portion $\varepsilon p_{g}$ for newly incorporated level *h* and a portion $\left(1-\varepsilon \right)p_{g}$ for level *g*. Thus

$$\begin{array}{cc} H^{\prime }\left(X\right) & =\left[H\left(X\right)+p_{g}\log\left(p_{g}\right)\right]-\varepsilon p_{g}\log\left(\varepsilon p_{g}\right)-\left(1-\varepsilon \right)p_{g}\log\left(\left(1-\varepsilon \right)p_{g}\right)\\ & =\left[H\left(X\right)+p_{g}\log\left(p_{g}\right)\right]-\varepsilon p_{g}\left(\log\left(\varepsilon \right)+\log\left(p_{g}\right)\right)-\left(1-\varepsilon \right)p_{g}\left(\log\left(1-\varepsilon \right)+\log\left(p_{g}\right)\right)\\ & =\left[H\left(X\right)+p_{g}\log\left(p_{g}\right)\right]-\varepsilon p_{g}\log\left(\varepsilon \right)-\varepsilon p_{g}\log\left(p_{g}\right)-\left(1-\varepsilon \right)p_{g}\log\left(1-\varepsilon \right)-\left(1-\varepsilon \right)p_{g}\log\left(p_{g}\right)\\ & =H\left(X\right)-\varepsilon p_{g}\log\left(\varepsilon \right)-\left(1-\varepsilon \right)p_{g}\log\left(1-\varepsilon \right)\\ & >H(X). \end{array}$$

The term $-p_{g}\left[\varepsilon \log\left(\varepsilon \right)+\left(1-\varepsilon \right)\log\left(1-\varepsilon \right)\right]$ is a positive value representing the increase in entropy when a new level of grey is incorporated in the region. This completes the proof. □

The smallest possible frequency for any level of grey in a region of *m* pixels is 1/m as mentioned in the Lemma. In practice, ε may be larger than 1/m; this occurs when more than one pixel is assigned the new grey level *h*. It is easy to show that ε=0.5 would yield a maximum increase in entropy at current iteration; however, according to the method proposed below (next subsection), entropy increases incrementally as the algorithm iterates.

### 3.4. Proposed Method Using Multiscale Top-Hat Transform

The proposed method is based on the multiscale top-hat transform. This method employs two structuring elements in the top-hat transform to improve its performance. The proposed method improves the image in terms of detail, contrast and mean brightness conservation. The infrared image enhancement algorithm initially uses the following parameters: The original image *I*, the number of iterations *n* in a range i∈{1,2,…,n}, n>1; and two structuring elements *G* and $G^{\prime }$.

Multiple Brightness (MB) and Multiple Darkness (MD) areas will be obtained by top-hat transform as follows:

(11) $$MB_{i}=I-\left(\left(I⊖G_{i}\right)⊕G_{i}^{\prime }\right),$$

where $MB_{i}$ is the *i*-scales of brightness extracted from the image, and $G_{i}$ and $G_{i}^{\prime }$ will grow in each iteration. $G^{\prime }$ will always be greater than or equal to *G*.

(12) $$MD_{i}=\left(\left(I⊕G_{i}\right)⊖G_{i}^{{}^{\prime }}\right)-I,$$

where $MD_{i}$ are the *i*-scales of darkness extracted from the image.

The Subtractions of the Neighboring Bright Scales (SNBS) are then calculated. This operation is expressed as follows:

(13) $$SNBS_{i-1}=\left\{\begin{array}{c} MB_{i}-MB_{i-1},\;\mathrm{to}\;i=2\\ MB_{i}-SNBS_{i-2},\;\mathrm{to}\;i>2 \end{array}\right.$$

where $SNBS_{i-1}$ are the (i−1)-differences of the neighboring brightness scales obtained from the image.

Similarly, the Subtractions of the Neighboring Dark Scales (SNDS) are calculated. This operation is expressed as follows:

(14) $$SNDS_{i-1}=\left\{\begin{array}{c} MD_{i}-MD_{i-1},\;\mathrm{to}\;i=2\\ MD_{i}-SNDS_{i-2},\;\mathrm{to}\;i>2 \end{array}\right.$$

where $SNDS_{i-1}$ are the (i−1)-differences of the neighboring dark scales obtained from the image.

The Sum of all the brightness (SMB and SSNBS) and darkness (SMD and SSNDS) values obtained in the multiscale process are then calculated as follows:

(15) $$SMB=\sum_{i=1}^{n}MB_{i},$$

(16) $$SMD=\sum_{i=1}^{n}MD_{i},$$

(17) $$SSNBS=\sum_{i=1}^{n-1}SNBS_{i-1},$$

(18) $$SSNDS=\sum_{i=1}^{n-1}SNDS_{i-1}.$$

Finally, the image enhancement $\left(I_{E}\right)$ will be obtained as follows:

(19) $$I_{E}=I+\omega \times \left(SMB+SSNBS\right)-\omega \times \left(SND+SSNDS\right),$$

where ω∈[0,1] is a weighting factor or regulator of the bright and dark areas.

The TII enhancement process is described in the following Algorithm 1.

**Algorithm 1** Proposed method for TII Enhancement**Input:***I*, *G*, $G^{\prime }$, *n*, ω **Output:**$I_{E}$*(Enhanced image)*  *Initialization*: *G*, $G^{\prime }$ 1: **for**i=1 to *n*
**do**
 2: *Calculation of top-hat transform.*    $MB_{i}=I-\left(\left(I⊖G_{i}\right)⊕G_{i}^{\prime }\right)$ (Equation (11))
    $MD_{i}=\left(\left(I⊕G_{i}\right)⊖G_{i}^{\prime }\right)-I$ (Equation (12))
 3: *Calculation of subtractions from neighboring scales, obtained through the top-hat transform. The top-hat is subtracted with the previous difference, from the first subtraction of the first neighboring top-hat.*
$$SNBS_{i-1}=\left\{\begin{array}{c} MB_{i}-MB_{i-1},\;\mathrm{to}\;i=2\\ MB_{i}-SNBS_{i-2},\;\mathrm{to}\;i>2 \end{array}\right.\;(\mathrm{Equation}\;(13))$$
$$SNDS_{i-1}=\left\{\begin{array}{c} MD_{i}-MD_{i-1},\;\mathrm{to}\;i=2\\ MD_{i}-SNDS_{i-2},\;\mathrm{to}\;i>2 \end{array}\right.\;(\mathrm{Equation}\;(14))$$
 4: **end for**
 5: *Calculation of the maximum values of all the multiple scales obtained.*
  $SMB=\sum_{i=1}^{n}MB_{i}$ (Equation (15))
  $SMD=\sum_{i=1}^{n}MD_{i}$ (Equation (16))
  $SSNBS=\sum_{i=1}^{n-1}SNBS_{i-1}$ (Equation (17))
  $SSNDS=\sum_{i=1}^{n-1}SNDS_{i-1}$ (Equation (18))
 6: *TII enhancement calculation.The contrast enhancement calculation consists of adding the results of the multiple bright scales to the original image and subtracting the results of the multiple dark scales*.
  $I_{E}=I+\omega \times \left(SMB+SSNBS\right)-\omega \times \left(SND+SSNDS\right)$ (Equation (19))
 7: **return**
$I_{E}$

## 4. Results and Discussion

**Figure 4:**
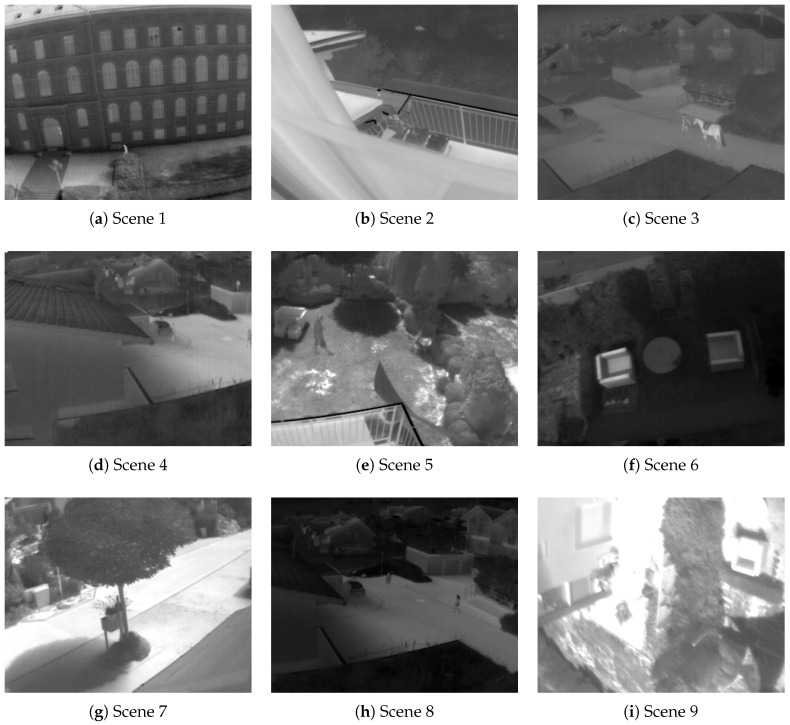
Examples of scenes from the database.

Experiments were performed by randomly selecting 450 TII of 324×256 from a public repository [50]. We analyzed 9 different scenes of 50 images each one. Images were captured with an infrared thermal camera FLIR Tau 320 with a resolution of 324×256 pixels. Images in the database are 8-bit and 16-bit. The database has no radiometric data. Tests were performed on the 8-bit images. Figure 4 shows the scenes. The computer used has the following features: Pentium Dual-Core 2.3 GHz processor, RAM 4GB, HD 1TB, and the operating system used was Windows 7.

In order to test the performance of the proposed method, we considered three different experiments:

- In the first part (Section 4.1) we perform a parameter adjustment to find good parameter values that maximize the entropy of the output image after applying the proposed method.
- Then, in the second part (Section 4.2) we analyze the proposed method per iteration and compare its performance with Multiscale Morphological Infrared Image Enhancement (MMIIE) (mathematical morphology-based multiscale approach) [4].
- Finally, in the last part (Section 4.3), we apply the proposed method and compare the results achieved with the proposed techniques with the following competitive methods from the literature: HE, Contrast Limited Adaptive Histogram Equalization (CLAHE) [51], the method of Kun Liang et al. [6] called IRHE2PL for infrared images, and the MMIIE method for infrared images.

### 4.1. Parameter Tuning

In this section, the goal is to find a good combination of values of the parameters of ω and the number of iterations *n*. Parameter ω has real values. As we cannot perform tests for all real values, we take a selection criteria for values that we consider representative to get good outcomes for ω and *n*. The search for more optimal values of these parameters could be approached in future work as an optimization problem. For this experiment we applied the proposed method in the selected dataset. Since we are seeking to optimize the entropy of the resulting image, we use such Equation (1) as evaluation metrics.

The parameter values of the proposed method are presented in Table 1. As shown, we tested different values of the number of iterations *n* and *w*. For *n*, we changed the value from 2 to 10. No larger values were considered because the larger the value is, the more the image becomes distorted. The parameter *w* was changed in the range of [0,1] in increments of 0.05. A value of 0 gives as result the original image. The initial structuring elements *G* and $G^{\prime }$ are squares of 3×3 and 15×15, respectively. In each iteration the two structuring elements will side increase in sizes of two.

Table 2 presents the results obtained. Each column refers to the corresponding iteration *n* while each row corresponds to a different value of ω. Higher results are highlighted in bold. The highest result is achieved with n=8 and ω=0.35. It is worth stressing that the entropy increases when the iteration rises its value until the local optima and from there, values start to decrease.

**Figure 5:**
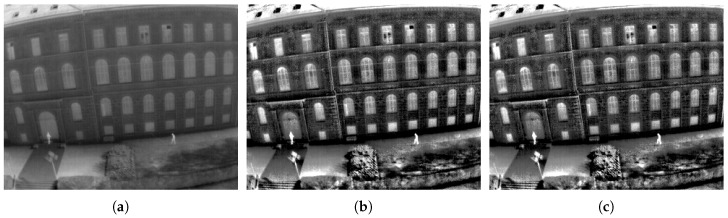
Visual results obtained with the proposed method and the configuration of ω and *n*. (**a**) Original TII with E=6.7893; (**b**) TII enhancement with proposed method with ω=0.35, n=8 and E=7.4696; (**c**) TII enhancement with proposed method with ω=0.45, n=7 and E=7.4467.

In Figure 5 we can see that images of the same scene with similar entropy, but with different configurations of ω and *n*, get similar visual results. For the other experiments, we select the configuration that has the best average.

### 4.2. Performance of Proposed Method per Iteration

In this section we compare the results per iteration between proposed method and MMIIE method, using the 450 infrared thermal images. We compare the performance of the entropy (Equation (1)) and with the following metrics:

- The *Standard Deviation* (SD), which quantifies the global contrast of the infrared images, is defined as [16]:
  (20) $$SD\left(I\right)=\sqrt{\sum_{k=0}^{L-1}{\left(k-A\left(I\right)\right)}^{2}\times p\left(k\right)},$$
  where *k* is the pixel value of the image *I*, L−1 is the maximum gray level, the average intensity of the image is represented by A(I), and p(k) is the probability of occurrence of the value *k*. If $SD(I_{E})$ is greater than SD(I), then there is contrast enhancement.
- The metric adopted to measure the signal-to-noise ratio of an image is the PSNR.
  Given the original infrared image *I* and the infrared image with enhancement $I_{EN}$ where the size of the images is M×N, the PSNR between *I* and $I_{EN}$ is given by [30]:
  (21) $$PSNR\left(I,I_{E}\right)=10\times log_{10}\frac{{\left(L-1\right)}^{2}}{MSE(I,I_{E})}.$$
  The *Mean Squared Error* (MSE) is defined as:
  (22) $$MSE\left(I,I_{E}\right)=\frac{1}{M\times N}\sum_{u=0}^{M-1}\sum_{v=0}^{N-1}{\left(I\left(u,v\right)-I_{EN}\left(u,v\right)\right)}^{2}.$$
- The *Absolute Mean Brightness Error* (AMBE) [11], which quantifies the conservation of the mean brightness of the processed image, is given by:
  (23) $$AMBE\left(I,I_{E}\right)=\left|A\left(I\right)-A\left(I_{E}\right)\right|,$$
  where *I* and $I_{E}$ represent the input infrared image and the image enhancement, respectively, A(I) and $A(I_{E})$ represent the mean brightness of the input infrared image and the image enhancement. The lower the AMBE value, the better the mean brightness of the image is preserved.
- The linear blur index γ [4] is used to measure the performance of the infrared image enhancement. It is defined as follows:
  (24) $$\gamma \left(I\right)=\frac{2}{M\times N}\sum_{u=1}^{M}\sum_{v=1}^{N}min\left\{p_{uv},\left(1-p_{uv}\right)\right\},$$
  (25) $$p_{uv}=sin\left[\frac{\pi }{2}\times \left(1-\frac{I(u,v)}{L-1}\right)\right].$$
  where M×N is the size of the infrared image. I(u,v) is the gray pixel value (u,v). L−1 is the maximum gray value of *I*. The performance of the algorithm is better if the value of γ is small.

Following the recommendations of Bai [4], we set the following parameter values for MMIIE method. The number of iterations *n* was set to 10, the weights to $w_{1}=0.6$, $w_{2}=w_{3}=1.5$, and the initial structuring element *B* was fixed to 3×3. For the proposed strategy and taking into account the parameter tuning, we fixed *w* to 0.35 and considered the same parameter values as in the previous experiment; n=10 and *G* and $G^{\prime }$ to 3×3 and 15×15 respectively. In each iteration the two structuring elements will increase in sizes of two. The average entropy of the 450 original thermal infrared images is E=6.5924. The proposed method and MMIIE was implemented using ImageJ [49].

In Table 3 it can be seen the results on average on each iteration for the 450 images with the proposed method and MMIIE method. Results in bold refer to the best results by iteration. According to the results obtained we can say that the proposed method outperforms MMIIE method in four of the five evaluation measures on every iteration, while MMIIE method achieves better results with γ. Therefore, on average, the proposed method provides better contrast enhancement and signal-to-noise ratio and higher level of detail. Furthermore, it also preserves better the brightness. On the other hand MMIIEE method provides better blur effect. The computational time of the proposed method is higher than that of the MMIIE method, but the proposed method obtains better results in fewer iterations (lower *n*).

**Figure 6:**
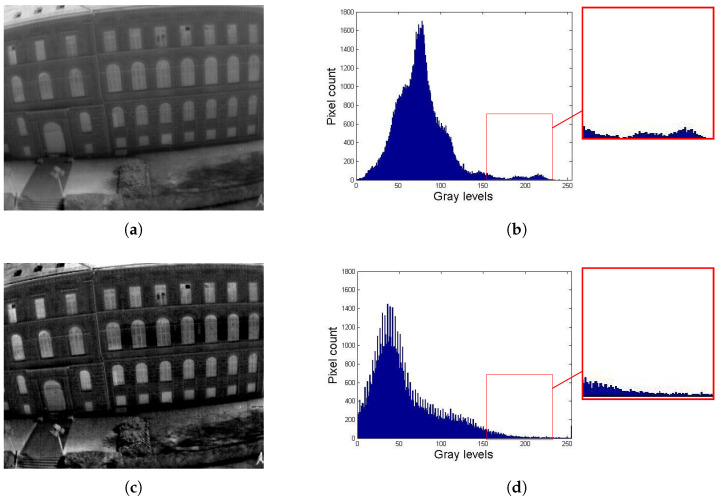

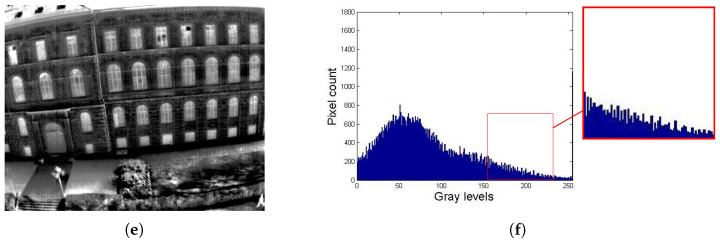
Example of a thermal infrared imaging (TII) to compare the proposed method and Multiscale Morphological Infrared Image Enhancement (MMIIE) method. (**a**) Original TII with E=6.8387 and SD=31.8204; (**b**) Histogram of the original image; (**c**) TII enhanced with the MMIIE method with E=6.8619, SD=42.5731, and AMBE=24.1018; (**d**) Histogram of the image enhanced with MMIIE; (**e**) TII enhanced with the proposed method with E=7.3629, SD=58.8730, and AMBE=2.1848; (**f**) Histogram of the image enhanced with the proposed method.

Figure 6 presents an example image from the dataset with its corresponding histogram. The original image and its histogram is shown in Figure 6a,b respectively. Figure 6c is the resulting image after applying MMIIE method and Figure 6e when applying the proposed method. In both cases we have selected the iteration in which the entropy is maximum. It can be observed that the proposed method presents a better redistribution of intensity levels in bright areas and has fewer peaks according to its histogram (Figure 6d,f. It therefore leads to a better level of detail and contrast.

### 4.3. Comparison of the Performance of the Proposed Method with State of the Art Methods

In this section we compare the proposed method with popular methods from the literature. The methods used in this section are HE, CLAHE, IRHE2PL [6], and MMIIE. In this part we analyze two results. First we are interested in knowing the percentage of images that are enhanced compared to the original using each method. Then, we analyze the performance of each method on each different scene.

The parameters of the various methods are as follows. For CLAHE, the method was implemented with the MATLAB program, using its default values. For the IRHE2PL method the parameters are described in [6]. For MMIIE method we set the number of iteration *n* to 9 and the weights to $w_{1}=0.6$, $w_{2}=w_{3}=1.5$. Finally, the initial structuring element *B* was fixed to 3×3 square. Results for this method use the number of iterations that maximize the entropy. Finally, for the proposed method we selected the best combination found in the first part. In this case n=8, ω=0.35, and the initial structuring elements *G* and $G^{\prime }$ are squares of 3×3 and 15×15, respectively. The HE, IRHE2PL, MMIIE methods and the proposed method were implemented using the ImageJ library [49].

Table 4 shows the percentage of images that have been enhanced in terms of contrast. An image is considered improved if the value of the standard deviation of the processed image is greater than the original image. As we can see, the proposed method enhances the contrast of the 450 TII. HE has a very high value with 98.89%, followed by IRHE2PL method, CLAHE method, and MMIE method with 90.22%, 82.67%, and 47.56%, respectively.

#### 4.3.1. Analysis of Methods by Scenes

The performance of each method in each scene is presented in Table 5. For each scene the first row refers to the original image followed by the different methods used in this study. The two best values for each metric are highlighted in bold. Results of each scene are presented are averaged over all the images that belong to such scene. At the end the average over all images is also presented.

Based on the averages of the metrics *E*, *SD*, *PSNR*, *AMBE*, and γ we can conclude that:

- *E* metric: The CLAHE method and the proposed method are the methods that have the best performance in terms of entropy for scenes 1 to 8. However, in scene 9 the CLAHE and MMIIE methods have the best results.
- *SD* metric: The HE, CLAHE, IRHE2PL methods and the proposed method enhance the contrast of the TII in the 9 scenes. The MMIIE method did not enhance the contrast of scenes 2, 4, 7, 8, and 9. The HE method is the best performing method for all scenes and the proposed method is in second place.
- *PSNR* metric: The methods that produce the less distortion to TII are the IRHE2PL, the proposed method, and CLA*HE*.
- *AMBE* metric: For all scenes, the best method in regards to maintaining the average brightness is the proposed method.
- γ metric: The MMIIE method and the proposed method present the best results in terms of blurring.

**Figure 7:**
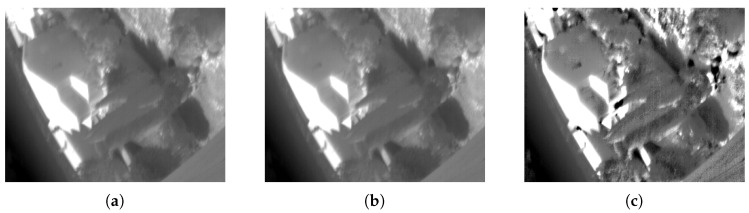
(**a**) Original TII 449.png, E=7.2210, SD=53.1827; (**b**) TII enhanced with IRHE2PL method, E=7.2058, SD=53.3164, and PSNR=48.0940 and (**c**) TII enhanced with the proposed method, E=7.2411, SD=62.8887, and PSNR=20.6578.

In the results of scenes 7 and 9 we can see that the IRHE2PL method does not improve the entropy of the image, but has a higher *PSNR* than the proposed method. This is because IRHE2PL generates an image very similar to the original image. Figure 7 shows that the TII (scene 9) enhanced with the IRHE2PL method (Figure 7b) is very similar to the original image (Figure 7a), contrary to the proposed method, which improves the entropy and contrast of the image (Figure 7c).

#### 4.3.2. General Analysis of Methods

In general none of the methods presented outperforms the other techniques in all evaluation criteria. In almost all cases, the proposed method is the strategy that achieves the best performance in entropy, contrast, and *AMBE*. Therefore, it is the one that provides better details and keeps a better brightness. HE is the algorithm that yields higher contrast according to its good performance, in all cases, with *SD*. The CLAHE method performs the best in entropy, so it preserves the details better. The best signal-to-noise ratio is achieved by the IRHE2PL method and, so, it is the one that provides the lowest distortion. Finally, the MMIIE method improves the blur effect in all cases. The proposed method provides the best values in AMBE in almost all cases and in *E*, *SD*, *PSNR*, and γ results are very competitive since it achieves high values that are close to the best value in many cases.

**Figure 8:**
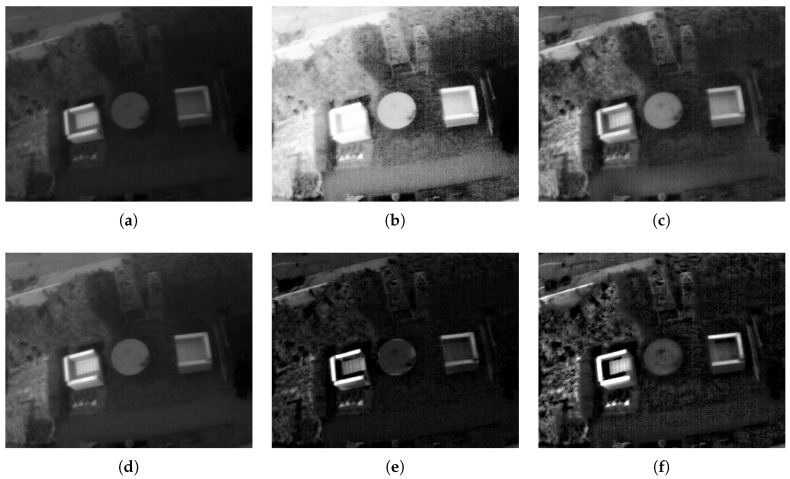
An example of comparison of a TII with a dark background. (**a**) Original TII, (**b**) TII enhanced with HE method, (**c**) TII enhanced with Contrast Limited Adaptive Histogram Equalization (CLAHE) method, (**d**) TII enhanced with IRHE2PL method, (**e**) TII enhanced with MMIIE method, and (**f**) TII enhanced with the proposed method.

**Figure 9:**
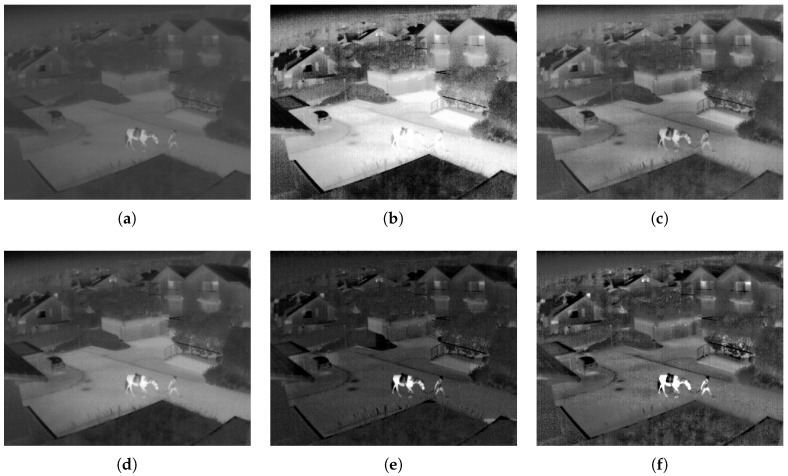
This example shows a TII with a dark background and semi-bright objectives. (**a**) original TII, (**b**) TII enhanced with HE, (**c**) TII enhanced with CLAHE, (**d**) TII enhanced with IRHE2PL, (**e**) TII enhanced with MMIIE, and (**f**) TII enhanced with the proposed method.

Now we can see a couple of examples in Figure 8 and Figure 9. For each image enhancement method, images were selected so that each method achieved the highest entropy value. In Figure 8 represents a patio scene images with Figure 8a the original image. The results of HE is in Figure 8b and it presents an excess of brightness. In Figure 8c the CLAHE method makes a moderate enhancement to the image. However, in Figure 8d the IRHE2PL method does not enhance the contrast of the TII. In Figure 8e the MMIIE method adds distortion to TII. Finally, in Figure 8f the proposed method enhances the contrast and improves the details of the TII. The second example, which represents a person with a horse, is in Figure 9. The analysis is similar to the previous case. Therefore we can emphasize the good performance of the proposed technique.

**Figure 10:**
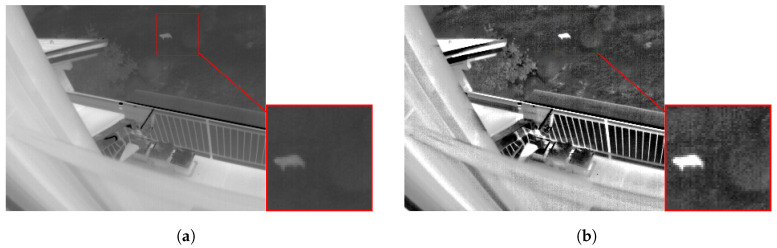
TII with improved contrast and detail, (**a**) the Original TII with E=6.9334 and SD=58.24 and (**b**) the TII enhanced with the proposed method with E=7.5783 and SD=70.7615

Finally, in Figure 10 we analyze the level of detail of the resulting image before and after applying the proposed strategy. Numerical results suggest that the level of details increase with the proposed method and this feature can be visually corroborated. For example, the resulting image in Figure 10b presents an excellent contrast, and a well-defined detail, which makes easier its identification.

## 5. Conclusions

In this work we have introduced an iterative contrast enhancement method for TII. This approach is based on multiscale top-hat transform that improves the entropy of images, which implies an improvement in the level of detail of the resulting image. Furthermore, the proposed method not only improves the entropy but also preserves the brightness and enhances contrast.

The proposed method was compared with state of the art algorithms and has proved to be competitive. It is noteworthy that the proposed method is the only algorithm that improved the original image for all input images in terms of contrast.

Visually, the resulting image after applying the proposed method presents a higher quality than the original image. This result is consistent with the performance of the algorithm. This proposed method could be very useful for infrared thermal image analysis, object recognition, people tracking, and other applications based on infrared thermal images. 

## Figures and Tables

**Table 1:** Parameter values for the parameter tuning experiment.

Parameter	Value(s)
*n*	[2,10]
ω	[0,1]
*G*	3×3
$G^{\prime }$	15×15

**Table 2:** Entropy values of the enhanced thermal infrared imaging (TII) obtained by the proposed method with parameters ω and *n*.

ω	*n*								
	2	3	4	5	6	7	8	9	10
0.05	6.5931	6.5962	6.6041	6.6188	6.6396	6.6656	6.6950	6.7271	**6.7607**
0.10	6.5971	6.6120	6.6394	6.6777	6.7224	6.7696	6.8174	6.8658	**6.9129**
0.15	6.6037	6.6315	6.6756	6.7326	6.7900	6.8502	6.9100	6.9653	**7.0099**
0.20	6.6145	6.6577	6.7190	6.7861	6.8548	6.9228	6.9848	7.0326	**7.0593**
0.25	6.6242	6.6821	6.7540	6.8316	6.9081	6.9788	7.0349	**7.0661**	7.0648
0.30	6.6293	6.6970	6.7790	6.8678	6.9498	7.0185	7.0633	**7.0702**	7.0348
0.35	6.6430	6.7217	6.8089	6.9025	6.9851	7.0475	**7.0740**	7.0519	6.9828
0.40	6.6518	6.7420	6.8398	6.9380	7.0181	7.0673	**7.0688**	7.0161	6.9169
0.45	6.6568	6.7545	6.8607	6.9637	7.0394	**7.0735**	7.0505	6.9706	6.8448
0.50	6.6806	6.7872	6.8957	6.9957	7.0596	**7.0726**	7.0225	6.9165	6.7668
0.55	6.6824	6.7928	6.9068	7.0085	**7.0647**	7.0610	6.9900	6.8619	6.6915
0.60	6.6914	6.8113	6.9314	7.0295	**7.0702**	7.0450	6.9516	6.8011	6.6111
0.65	6.6945	6.8201	6.9451	7.0404	**7.0682**	7.0249	6.9112	6.7406	6.5330
0.70	6.7066	6.8408	6.9655	7.0516	**7.0631**	7.0010	6.8676	6.6786	6.4556
0.75	6.7161	6.8588	6.9835	**7.0602**	7.0550	6.9745	6.8216	6.6152	6.3778
0.80	6.7221	6.8707	6.9979	**7.0639**	7.0433	6.9462	6.7747	6.5531	6.3032
0.85	6.7259	6.8791	7.0077	**7.0650**	7.0303	6.9167	6.7289	6.4929	6.2319
0.90	6.7309	6.8906	7.0183	**7.0641**	7.0140	6.8843	6.6802	6.4311	6.1612
0.95	6.7326	6.8959	7.0235	**7.0604**	6.9973	6.8515	6.6320	6.3702	6.0927
1.00	6.7791	6.9368	7.0460	**7.0610**	6.9780	6.8134	6.5783	6.3055	6.0210

**Table 3:** Results achieved with the proposed method and Multiscale Morphological Infrared Image Enhancement (MMIIE) method.

*n*	Proposed Method						MMIIE					
	*E*	*SD*	*PSNR*	*AMBE*	γ	Time (ms)	*E*	*SD*	*PSNR*	*AMBE*	γ	Time (ms)
2	**6.643**	**40.835**	**40.417**	**0.129**	0.332	2328	6.324	30.247	16.236	38.072	**0.194**	**454**
3	**6.722**	**41.969**	**33.564**	**0.286**	0.320	3752	6.447	32.069	16.176	37.775	**0.193**	**902**
4	**6.809**	**43.632**	**29.256**	**0.466**	0.311	6719	6.441	33.016	16.211	37.528	**0.192**	**1605**
5	**6.902**	**45.854**	**26.023**	**0.714**	0.301	10,629	6.519	34.438	16.204	37.193	**0.193**	**2759**
6	**6.985**	**48.556**	**23.509**	**1.107**	0.293	11,947	6.515	35.486	16.216	36.902	**0.194**	**4770**
7	**7.047**	**51.749**	**21.434**	**1.636**	0.286	16,429	6.570	36.798	16.177	36.580	**0.195**	**7187**
8	**7.074**	**55.353**	**19.693**	**2.299**	0.281	19,979	6.565	37.558	16.194	36.318	**0.196**	**9255**
9	**7.052**	**59.216**	**18.219**	**3.034**	0.275	20,176	6.612	38.453	16.178	36.093	**0.197**	**18,318**
10	**6.983**	**63.111**	**16.980**	**3.830**	0.268	21,527	6.604	39.017	16.201	35.884	**0.197**	**20,003**

**Table 4:** Contrast improvement percentage.

Methods	Percentage of Images Improved (%)
HE	98.89%
CLAHE	82.67%
IRHE2PL	90.22%
MMIIE	47.56%
Proposed method	100%

**Table 5:** Average of the assessments of the 9 scenes obtained by the methods.

	Methods	*E*	*SD*	*PSNR*	*AMBE*	γ
**Scene 1**	I	6.814	32.336	-	-	0.284
	HE	6.596	**73.420**	11.543	48.519	0.406
	CLAHE	**7.557**	50.808	15.984	24.259	0.401
	IRHE2PL	6.814	36.776	**29.603**	**7.065**	0.293
	MMIIE	6.910	43.573	**17.005**	25.392	**0.164**
	Proposed method	**7.418**	**60.136**	16.767	**1.887**	**0.273**
**Scene 2**	I	7.039	55.330	-	-	0.454
	HE	6.844	**73.364**	20.479	3.101	**0.400**
	CLAHE	**7.500**	53.721	**21.237**	**1.657**	0.488
	IRHE2PL	7.036	58.604	**33.310**	6.802	0.453
	MMIIE	7.038	45.486	13.085	48.345	**0.273**
	Proposed method	**7.601**	**69.425**	18.215	**1.534**	0.419
**Scene 3**	I	5.945	18.269	-	-	0.477
	HE	5.881	**73.063**	10.789	47.807	0.408
	CLAHE	**6.970**	32.154	19.839	18.275	0.485
	IRHE2PL	5.945	**42.197**	**20.011**	**13.270**	0.326
	MMIIE	6.133	20.900	17.288	32.819	**0.127**
	Proposed method	**6.826**	30.332	**23.205**	**0.136**	**0.273**
**Scene 4**	I	6.808	41.521	-	-	0.342
	HE	6.642	**73.148**	12.977	41.972	0.407
	CLAHE	**7.482**	48.135	18.560	19.816	0.422
	IRHE2PL	6.808	56.144	**22.617**	**11.306**	0.313
	MMIIE	6.848	35.941	16.106	33.095	**0.149**
	Proposed method	**7.566**	**56.723**	**19.019**	**1.328**	**0.312**
**Scene 5**	I	7.052	40.839	-	-	0.356
	HE	6.901	**73.319**	14.793	32.298	0.404
	CLAHE	**7.620**	50.630	**17.786**	15.352	0.433
	IRHE2PL	7.048	45.807	**32.631**	**11.444**	**0.307**
	MMIIE	7.025	44.123	15.660	34.956	**0.173**
	Proposed method	**7.505**	**61.669**	17.599	**2.584**	0.317
**Scene 6**	I	6.272	24.626	-	-	0.152
	HE	6.158	**72.882**	8.091	86.636	0.408
	CLAHE	**7.200**	**42.379**	16.477	31.714	0.263
	IRHE2PL	6.272	37.308	**21.316**	17.733	0.179
	MMIIE	6.035	28.802	**21.602**	**15.389**	**0.048**
	Proposed method	**6.702**	40.545	20.893	**2.284**	**0.114**
**Scene 7**	I	6.990	67.015	-	-	0.348
	HE	6.783	**73.516**	18.535	19.921	0.398
	CLAHE	**7.548**	66.298	19.828	**7.159**	0.400
	IRHE2PL	6.987	75.173	**33.982**	14.009	**0.295**
	MMIIE	7.125	53.062	12.017	54.459	0.327
	Proposed method	**7.204**	**75.462**	**19.735**	**1.405**	**0.323**
**Scene 8**	I	6.219	28.522	-	-	0.237
	HE	6.131	**72.805**	8.042	89.033	0.409
	CLAHE	**7.134**	41.543	16.828	31.090	0.309
	IRHE2PL	6.219	**62.031**	11.319	60.173	0.334
	MMIIE	5.952	23.883	**21.792**	**14.567**	**0.077**
	Proposed method	**6.589**	37.486	**23.014**	**2.426**	**0.118**
**Scene 9**	I	6.191	53.458	-	-	0.448
	HE	6.001	**80.909**	13.975	41.396	**0.329**
	CLAHE	**6.459**	59.638	**19.045**	13.075	0.440
	IRHE2PL	6.188	**67.644**	**30.537**	**10.979**	0.386
	MMIIE	**6.438**	50.305	11.052	65.813	0.433
	Proposed method	6.254	66.401	18.794	**7.105**	**0.378**
